# Intraocular lens implantation in unilateral congenital cataract with minimal levels of persistent fetal vasculature in the first 18 months of life

**DOI:** 10.1186/2193-1801-3-361

**Published:** 2014-07-16

**Authors:** Toshihiko Matsuo

**Affiliations:** Department of Ophthalmology, Okayama University Medical School and Graduate School of Medicine, Dentistry, and Pharmaceutical Science, 2-5-1 Shikata-cho, Okayama City, 700-8558 Japan

**Keywords:** Congenital cataract, Persistent fetal vasculature (PFV), Persistent hyperplastic primary vitreous (PHPV), 25-Gauge vitrectomy, Surgery, Ultrasound examination, Lensectomy, Anterior vitrectomy, Posterior capsulotomy, Intraocular lens implantation

## Abstract

**Purpose:**

To describe the incidence of unilateral congenital cataract associated with minimal (ultrasonically undetectable) levels of persistent fetal vasculature in the first 18 months of the life and to report surgical methods for intraocular lens implantation, using 25-gauge vitrectomy system.

**Methods:**

Retrospective review was made on 16 consecutive patients with bilateral or unilateral congenital cataract in the first 18 months of the life who underwent surgery at Okayama University Hospital after the introduction of the 25-gauge vitrectomy system from October 2005 to March 2013. As the standard of care at this hospital in the study period, intraocular lenses were not implanted in children with bilateral cataract while intraocular lenses were implanted in those with unilateral cataract.

**Results:**

Ten children with bilateral cataract underwent lensectomy in both eyes with a 25-gauge vitreous cutter under irrigation with a 25-gauge infusion cannula, inserted from two side ports at the corneal limbus. Six children with unilateral cataract underwent intraocular lens implantation and posterior capsulotomy after lens aspiration from limbal side ports. No patient showed vitreous abnormalities on ultrasound examinations before the surgery. At the surgery, all 10 children with bilateral cataract showed no additional abnormalities. In contrast, 3 children with unilateral cataract at the age younger than 12 months showed white fibrous tissue in the anterior vitreous integrated with the posterior lens capsule while the other 3 children with unilateral cataract at the age from 12 to 18 months did not have vitreous abnormalities. The fibrous tissue was cut together in the process of posterior capsulotomy from a 25-gauge trocar inserted at 1.5 mm posterior from the corneal limbus.

**Conclusions:**

Unilateral congenital cataract in the first 12 months of the life has a high incidence for the association with anterior type of persistent fetal vasculature which could not be detected by preoperative ultrasound examinations. Intraocular lens implantation was technically feasible in unilateral cataract with or without minimal levels of persistent fetal vasculature in the first 18 months of the life.

## Background

Congenital cataract occurs bilaterally or unilaterally. Bilateral cataracts are familial, or associated with systemic syndromes, or caused by intrauterine episodes such as maternal rubella infection and corticosteroid medications (Apple et al. 2000; Lambert and Drack 1996; Lloyd et al. 1992; Rahi et al. 2000, Rahi et al. 2001; Taylor 1998; Wirth et al. 2002). The extent of lens opacity is usually similar between both eyes. In contrast, unilateral cataracts are sporadic and sometimes associated with other anomalies such as persistent hyperplastic primary vitreous (Morrison et al. 2011; Mullner-Eidenbock et al. 2004; Vasavada et al. 2012). The persistent hyperplastic primary vitreous (PHPV) is the presence of fetal hyaloid vascular system or its remnant in the vitreous cavity at birth (Dass and Trese 1999; Goldberg 1997; Haddad et al. 1978; Hunt et al. 2005; Peyman et al. 1976; Pollard 1997; Reese 1955). The situation is also called persistent fetal vasculature (Goldberg 1997), which has been coined as a new term and advocated to be used in place of PHPV. The persistent fetal vasculature or PHPV is classified largely into two types, posterior type and anterior type, based on the clinical pictures.

The standard care for congenital cataract is lensectomy and visual rehabilitation to avoid or treat form-vision deprivation amblyopia (Lambert and Drack 1996; Lloyd et al. 1992; Taylor 1998). Pars plicata lensectomy by a vitrectomy system has replaced cataract surgery from a corneoscleral incision and has become a standard surgical method for total lensectomy, including cortical aspiration and capsulectomy. More recently, intraocular lens implantation has been challenged in congenital cataract at the younger age (Hiles 1984; O’Keefe et al. 2001; Swamy et al. 2007), and thus, cataract surgery from the corneoscleral incision with side ports at the corneal limbus has returned to the stage.

The 25-gauge vitrectomy system would be exceptionally suitable for vitrectomy and lensectomy through side ports at the corneal limbus. In this study, I reported the incidence of unilateral congenital cataract associated with minimal (ultrasonically undetectable) levels of persistent fetal vasculature in the first 18 months of the life in the series of 16 consecutive patients with bilateral or unilateral congenital cataract, and described surgical methods for intraocular lens implantation, using the 25-gauge vitrectomy system, in a systematic way.

## Results

No patient in this study, involving 16 consecutive patients with bilateral or unilateral congenital cataract in the first 18 months of the life, showed vitreous abnormalities on ultrasound examinations before the surgery. Ten patients had bilateral cataract while 6 patients showed unilateral cataract. The size of the eyes with unilateral cataract in 6 patients was basically the same as that of the contralateral healthy eyes.

**Figure 1:**
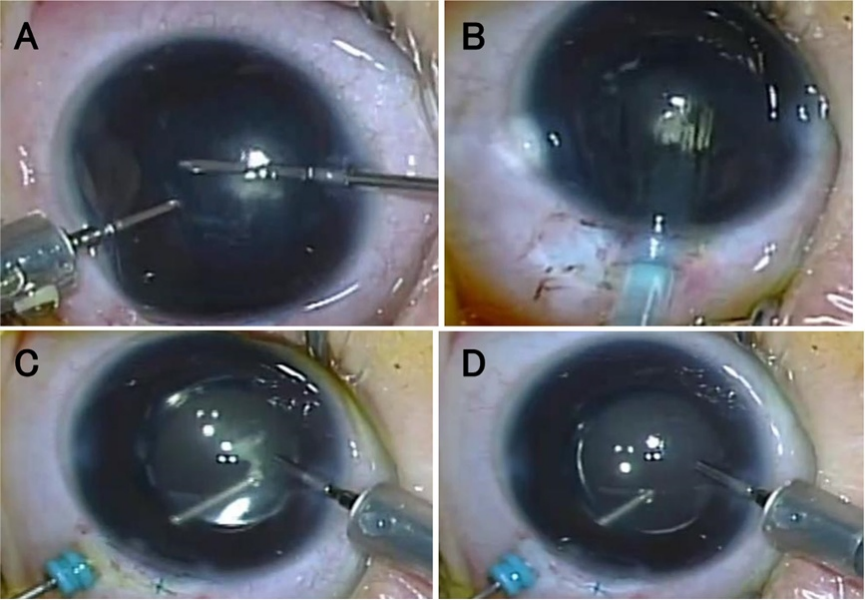
**Images taken from surgical video in a 7-month-old boy (Case 16).** Cataract surgery with 25-gauge vitrectomy system. Anterior capsulectomy and aspiration with a 25-gauge vitreous cutter, under irrigation with a 25-gauge cannula, through two side ports at the corneal limbus **(A)**. Injector-preloaded acrylic intraocular lens implantation through a corneoscleral tunnel incision **(B)**. Posterior capsulotomy, together with excision of white fibrous tissue of persistent fetal vasculature, by a vitreous cutter through a trocar at the pars plicata, under the irrigation by an infusion cannula inserted from a limbal side port **(C)**. Anterior vitrectomy is also done **(D)**.

At the surgery, all 10 children with bilateral cataract showed no additional abnormalities other than cataract in itself. In contrast, 3 children with unilateral cataract at the age younger than 12 months showed white fibrous tissue in the anterior vitreous, integrated with the posterior lens capsule, while the other 3 children with unilateral cataract at the age from 12 to 18 months did not have vitreous abnormalities (Table 1). The fibrous tissue was cut together in the process of posterior capsulotomy from a 25-gauge trocar inserted at the pars plicata, 1.5 mm posterior from the corneal limbus (Figure 1).

**Table 1:** **Summary of 16 consecutive patients with congenital cataract who underwent surgery in the first 18 months of life**

Case No./Sex	Laterality	Age at surgery	Surgery	Age at final visit	Follow-up period	Best-corrected visual acuity at final visit Right eye/Left eye	Other features
1/Male	Bilateral	2 mo.	Bil. lensectomy	8 yr. 11 mo.	105 mo.	0.7/0.5	Familial, Brother of Case 15, Father: aphakic bilaterally
2/Male	Bilateral	9 mo.	Bil. lensectomy	8 yr. 5 mo.	92 mo.	0.4 with both eyes open	Mental delay
3/Female	Bilateral	3 mo.	Bil. lensectomy	7 yr. 11 mo.	92 mo.	0.7/0.9	
4/Male	Right	15 mo.	Rt. IOL implantation	8 yr. 8 mo.	89 mo.	1.0/1.0	
5/Male	Bilateral	3 mo.	Bil. lensectomy	6 yr. 3 mo.	72 mo.	0.3/0.4	Esotropia
6/Male	Bilateral	4 mo.	Bil. lensectomy	4 yr. 11 mo.	55 mo.	Unmeasurable	Mental delay
7/Male	Bilateral	16 mo.	Bil. lensectomy	6 yr. 7 mo.	79 mo.	1.2/1.0	Familial, Sister and father: aphakic bilaterally
8/Male	Bilateral	4 mo.	Bil. lensectomy	5 yr. 3 mo.	59 mo.	0.03/0.3	Infantile glaucoma, bilateral trabeculectomy
9/Male	Right	5 mo.	Rt. IOL implantation	4 yr. 11 mo.	59 mo.	0.02/1.0	Minimal level of anterior PFV
10/Female	Left	1 mo.	Lt. IOL implantation	2 yr.	23 mo.	Unmeasurable	Minimal level of anterior PFV
11/Female	Bilateral	4 mo.	Bil. lensectomy	3 yr.	32 mo.	Unmeasurable	Cleft and lip palate
12/Female	Right	17 mo.	Rt. IOL implantation	3 yr. 9 mo.	28 mo.	0.04/1.0	
13/Male	Bilateral	4 mo.	Bil. lensectomy	2 yr. 9 mo.	29 mo.	Unmeasurable	Hydrocephalus, macular atrophy, optic disc atrophy
14/Male	Left	15 mo.	Lt. IOL implantation	3 yr. 6 mo.	27 mo.	1.0/0.05	
15/Male	Bilateral	1 mo.	Bil. lensectomy	1 yr. 8 mo.	19 mo.	Unmeasurable	Familial, Brother of Case 1, Father: aphakic bilaterally
16/Male	Left	7 mo.	Lt. IOL implantation	1 yr. 7 mo.	12 mo.	Unmeasurable	Minimal level of anterior PFV

Bil., bilateral; Rt., right; Lt., left; IOL, intraocular lens; PFV, persistent fetal vasculature; yr., years; mo., months.

Bilateral lensectomy indicates lensectomy and anterior vitrectomy with 25-gauge vitreous cutter inserted from one side port at corneal limbus, under irrigation with 25-gauge infusion cannula placed at another side port. IOL implantation indicates circular anterior capsulectomy and lens aspiration with 25-gauge vitreous cutter, IOL implantation from corneoscleral tunnel incision, posterior capsulotomy and anterior vitrectomy from one trocar-guided scleral port at 1.5 mm from the limbus under irrigation from a limbal side port with 25-gauge infusion cannula.

All 10 children with bilateral aphakia after lensectomy in both eyes underwent retinoscopy to determine the refraction and began to wear glasses. The refraction with the glasses was adjusted at -2 diopters to aim at near fixation around 50 cm. The 6 children with unilateral intraocular lens implantation also underwent retinoscopy and began to wear glasses to correct the hyperopia and astigmatism. Occlusion with eye patches upon contralateral healthy eyes for 2-6 hours daily was started in most of children with unilateral intraocular lens implantation.

The best-corrected visual acuity in both eyes at the final visit was at acceptable levels in children with bilateral aphakia, except for one child who had infantile glaucoma concurrently (Table 1). In contrast, the best-corrected visual acuity in the eyes with intraocular lens implantation was basically poor, compared with the normal levels of visual acuity in the contralateral healthy eyes (Table 1). As an exception, one child (Case 4) with unilateral cataract who underwent intraocular lens implantation at the age of 15 months gained the visual acuity of 1.0 in both eyes (Table 1).

## Discussion

The goal of this study is to report technical feasibility of intraocular lens implantation in the first 18 months of the life and to reconfirm the incidence of anterior persistent fetal vasculature at a minimal level, complicated with congenital cataract, as described in the literature (Morrison et al. 2011; Mullner-Eidenbock et al. 2004; Vasavada et al. 2012). The standard surgical method for lensectomy in infants, described in textbooks and literatures, is pars plana vitrectomy, or more accurately, pars plicata vitrectomy, since the anterior-to-posterior width of the pars plana is narrow in infants. In case of implanting the intraocular lens, cataract surgery was done as usual as in adults, and then posterior capsulotomy with anterior vitrectomy was additionally performed through the pars plana or plicata (O’Keefe et al. 2001; Swamy et al. 2007). In the present study, we described, for the first time, a systematic surgical method for congenital cataract, using 25-gauge vitrectomy system.

The 25-gauge vitrectomy system provides surgical instruments which are suitable for the manipulation through side ports at the corneal limbus. A 25-gauge vitreous cutter is inserted from one side port and a 25-gauge infusion cannula is held at another side port to maintain the anterior chamber with continuous irrigation. Anterior circular capsulectomy, similar to continuous curvilinear capsulorrhexis, is easily accomplished with a vitreous cutter. The lens cortex in the capsular bag is then aspirated with a vitreous cutter in a cutter-off mode. Bimanual irrigation and aspiration system for cataract surgery can be used instead of the 25-gauge vitrectomy system. After the intraocular lens is implanted in the capsular bag from the corneoscleral incision on the superior side, a 25-gauge trocar is inserted directly and perpendicularly from the scleral surface under the irrigation to maintain the intraocular pressure with a 25-gauge cannula from the limbal side port. Only one trocar at the pars plicata to pars plana is enough to make posterior capsulotomy and anterior vitrectomy with the aid of irrigation from the limbal side port.

Surgical results for persistent fetal vasculature or PHPV in general have been well described in the literature (Alexandrakis et al. 2000; Dass and Trese 1999; Goldberg 1997; Haddad et al. 1978; Hunt et al. 2005; Peyman et al. 1976; Pollard 1997; Reese 1955; Soheilian et al. 2002). The persistent fetal vasculature is also a well-known cause of unilateral congenital cataract (Apple et al. 2000; Lambert and Drack 1996; Lloyd et al. 1992; Morrison et al. 2011; Mullner-Eidenbock et al. 2004; Rahi et al. 2000; Rahi et al. 2001; Taylor 1998; Wirth et al. 2002). In this context, lensectomy combined with vitrectomy is a standard treatment for unilateral cataract with persistent fetal vasculature. Intraocular lens implantation was performed in some cases (Morita et al. 2001; Morrison et al. 2011; Vasavada et al. 2012). In the presence of cataract, persistent fetal vasculature is detected usually by ultrasound examination at ophthalmology clinics. As a diagnostic procedure in case of unilateral cataract, intraocular imaging, such as ultrasound examination, computed tomographic scan or magnetic resonance imaging (Castillo et al. 1997), is mandatory to exclude retinoblastoma, Coats disease, and persistent fetal vasculature.

In the present series of patients, preoperative ultrasound examinations did not detect any abnormalities in the eyes. At the surgery after aspiration of the lens cortex, a white fibrous tissue, integrated with the posterior capsule, was noted as the anterior type of persistent fetal vasculature in 3 patients at the age younger than 12 months. In a previous report, minimal fetal vascular remnants were detected in all eyes with unilateral congenital cataract (Mullner-Eidenbock et al. 2004). In the present series of patients, however, 3 patients at the age of 12 to 18 months had no additional intraocular abnormalities, except for unilateral cataract. Therefore, minimal levels of anterior persistent fetal vasculature would underlie a large part of unilateral congenital cataract at the younger age, but could not explain all unilateral cataract as an underlying cause.

The intraocular lens implantation is now the standard of care in patients with cataract in the age of 2 years or older. In contrast, the intraocular lens implantation in the first 18 months of the life remains still controversial at moment (Kuhli-Hattenbach et al. 2008; Plager et al. 2011). In the present series of patients, I set a tentative standard of care at my hospital to implant the intraocular lens in unilateral cataract, but not to implant the lens in bilateral cataract, in the first 18 months of the life. Bilateral aphakia is managed by wearing glasses while unilateral aphakia requires contact lens correction. The contact lens management puts a huge burden on family members of patients both physically and mentally.

In the present study, the intraocular lens, designed for adults, although appearing rather large, could be fixed in the capsular bag in all 6 children. Furthermore, any complications, such as pupillary opacity and glaucoma, were not noted in the follow-up. Notwithstanding the absence of postoperative complications, the visual acuity in 3 children with intraocular lens implantation for unilateral cataract still remained worse at the final visit. This poor visual outcome would be attributed to poor compliance, not only with wearing glasses, but also with occlusion therapy (eye patching) to contralateral healthy eyes in the background of deep amblyopia. One child (Case 4) with intraocular lens implantation at the age of 15 months, exceptionally, gained good visual acuity in the operated-on eye in the follow-up. The unilateral cataract in this child would not be present from birth, but would have developed rather later in growth.

## Conclusions

This study showed technical feasibility of the 25-gauge vitrectomy system for congenital cataract surgery in the first 18 months of the life. The two main technical points are 1) that anterior capsulectomy and cortical aspiration are accomplished through two side ports at the corneal limbus, and 2) that posterior capsulotomy and anterior vitrectomy are done through a single pars plicata trocar under irrigation from a limbal side port after the intraocular lens implantation. This study also confirmed that unilateral congenital cataract, with no ultrasound abnormalities, in the first 12 months of the life, has the background of the minimal anterior type of persistent fetal vasculature. The residual presence of persistent fetal vasculature can be managed efficiently and safely by the present technique with the 25-gauge vitrectomy system.

## Methods

### Patients

Retrospective review was made on 16 consecutive patients with congenital cataract in the first 18 months of the life who underwent surgery at Okayama University Hospital after the introduction of the 25-gauge vitrectomy system from October 2005 to March 2013 (Table 1). The children were 12 boys and 4 girls. All children underwent surgery as soon as possible after the initial visit to treat form-vision deprivation amblyopia and to avoid its further deterioration. Exclusion criteria in this study were eyes with vitreous abnormalities such as persistent fetal vasculature, detected by ultrasound examinations, in addition to cataract. The age at the surgery ranged from 1 month to 17 months (mean, 6.8 months) and the follow-up period ranged from 12 months to 105 months (mean, 54.5 months). This study adhered to the tenets of the Declaration of Helsinki and was approved by the institutional review board (Ethics Committee of Okayama University Graduate School of Medicine, Dentistry, and Pharmaceutical Sciences) as a retrospective case-series study.

As the standard of care at this hospital in the study period, intraocular lenses were not implanted in children with bilateral cataract while intraocular lenses were implanted in those with unilateral cataract. Ten children with bilateral cataract underwent lensectomy in both eyes with a 25-gauge vitreous cutter under irrigation with a 25-gauge infusion cannula, inserted from two side ports at the corneal limbus. Six children with unilateral cataract underwent intraocular lens implantation and posterior capsulotomy after lens aspiration from limbal side ports (Table 1).

The power of the intraocular lens was calculated by the SRK/T formula with keratometric and axial length measurements obtained before the surgery. The goal of intraocular lens implantation was set at attaining a lower grade of hyperopia, the grade of which was based on the age of a child at the surgery (Plager et al. 2002; Kim and Plager 2009).

### Surgical Methods

Two side ports at the corneal limbus on the nasal and temporal side were made by a 20-gauge knife (Corneal/Scleral V-LANCE Knife, Alcon, Fort Worth, TX, USA). Under the continuous irrigation with a 25-gauge infusion cannula held at one side port, circular anterior capsulectomy was made by a 25-gauge vitreous cutter, and the lens cortex and nucleus were aspirated inside the capsular bag by a vitreous cutter in a changing mode for either cutting or aspiration (ACCURUS Surgical System and CONSTELLATION Vision System, Alcon). In case of total lensectomy, anterior and posterior capsulectomy and then anterior vitrectomy was performed from limbal side ports.

In case of intraocular lens implantation, the conjunctiva was cut at the limbus on the superior side and a corneoscleral tunnel incision with 2.4 mm width was made. An intraocular lens (foldable acrylic lens preloaded in an injector) was implanted in the capsular bag with the aid of hyaluronan which was later aspirated from side ports by a vitreous cutter in an aspiration mode.

Under the irrigation at one side port with a 25-gauge infusion cannula, a 25-gauge trocar was inserted perpendicularly to the scleral surface in the superotemporal quadrant of the pars plana to plicata junction at 1.5 mm from the corneal limbus. Posterior capsulotomy and anterior vitrectomy were done with a vitreous cutter. The trocar was removed and the scleral wound was sutured with 8-0 virgin silk. All surgeries were done by a single surgeon (T. M.) under general anesthesia.

### Visual Acuity Testing

The visual acuity at distant viewing was tested at the distance of 5 m with internationally standard Landolt-C charts. Children at the younger age were tested with Landolt-C cards at 2.5 m for distant viewing and the visual acuity at 2.5 m was converted to that presumed at the measuring distance of 5 m. The visual acuity at near viewing was tested at about 30 cm with a hand-held Landolt-C cards for near viewing. The intraocular pressure in children was measured with a hand-held tonometer (Icare TA01i, Icare Finland, Helsinki). The other standard ophthalmological examinations included hand-held or table-fixed autorefraction, hand-held slit-lamp biomicroscopy, and funduscopy.
